# Complete chloroplast genome sequences of *Impatiens macrovexilla* and *I. macrovexilla* var. *yaoshanensis* (Balsaminaceae)

**DOI:** 10.1080/23802359.2022.2119104

**Published:** 2022-09-15

**Authors:** Shikai Guan, Dayan Tao, Jinye Zhou, Haixia Yan, Qian Song, Shuming Luo

**Affiliations:** a Flower Research Institute, Guangxi Academy of Agricultural Sciences, Nanning, Guangxi, China; b Faculty of Agriculture and Environment, Plant Breeding Institute, University of Sydney, Cobbitty, NSW, Australia

**Keywords:** *Impatiens macrovexilla*, *Impatiens macrovexilla* var. *yaoshanensis*, chloroplast genome, genome assembly, phylogenetic tree

## Abstract

Both *Impatiens macrovexilla* and *I*. *macrovexilla* var. *yaoshanensis* have potential to be exploited as ornamental plants, despite some of their morphological differences. In the present study, the complete chloroplast genome sequences of the two taxa are reported for the first time, which could facilitate their infraspecies classification, and analyses of their evolution, phylogeny, and breeding potential. The chloroplast genomes of *I*. *macrovexilla* and *I*. *macrovexilla* var. *yaoshanensis* were 152,437 and 152,286 bp in size, respectively. Their total GC contents were 36.77 and 36.80%, respectively. Both genomes contained 88 protein-coding genes, 8 rRNA genes, and 37 tRNA genes. The phylogenetic analysis revealed that the two specimens clustered next to each other and were closely related to *I*. *alpicola*, *I*. *fanjingshanica*, and *I*. *piufanensis*, but relatively distant from *I*. *guizhouensis* and *I*. *pritzelii*.

Species classification within the genus *Impatiens* in Balsaminaceae has been taxonomically difficult, because the genus contains more than 1,000 species (Yu 2012; Tang et al. 2020). *Impatiens macrovexilla* (Chen 2000) and *I*. *macrovexilla* var. *yaoshanensis* Yu et al. 2007 are native species within the Northern Guangxi Zhuang Autonomous Region in China. Morphological differences of these two taxa include the ovate-orbicular leaf, lateral sepals, and obvious auricle of *I*. *macrovexilla* (Yu et al. 2007), as well as some molecular data variations (Zhou et al. 2021). Previous research has been limited mainly to the phylogeny and genetic diversity of the species (Zhao et al. 2014; Yu et al. 2016; Zhou et al. 2021). The purpose of this study was to sequence the whole chloroplast genome of these two taxa, and to compare their genetic relationships with other *Impatiens* species. Such results would be significant for their botanical classification, evolutionary history, phylogenetic analysis, and breeding potential.

Leaf tissue was sampled from Xingping (110.5599°E, 24.9308°N) for *I*. *macrovexilla*, and Jinxiu (110.1148°E, 23.9712°N) for *I*. *macrovexilla* var. *yaoshanensis*. These specimens were deposited in the Guangxi Academy of Agricultural Sciences (http://www.gxaas.net/, Shuming Luo, shumingluo17@163.com) under the voucher numbers GXAAS-Imp019 and GXAAS-Imp021. A modified CTAB method (Doyle and Doyle 1987) was used to extract the total genomic DNA, and then the DNA libraries were sequenced by Guangzhou Bio&Data Biotechnologies Co., Ltd. (Guangzhou, China) on the BGISEQ-500 platform with PE150 read lengths according to the manufacturer’s instructions. The raw reads were trimmed using Fastp (Chen et al. 2018), and the clean reads were assembled into the cp genome using *I. alpicola* (NC_053940.1) as the reference genome in NOVOPlasty (Dierckxsens et al. 2017). Finally, the resultant genome was annotated with CpGAVAS software (Liu et al. 2012). The sequences were aligned using MAFFT v.7.0 (Katoh and Standley 2013). A maximum likelihood phylogenetic tree of these two specimens and another 17 *Impatiens* species was constructed using FastTree version 2.1.10 using the generalized time-reversible model and the Shimodaira–Hasegawa test (Price et al. 2010). *Actinidia arguta* (Actinidiaceae) was used as an out-group, and all accessions in the tree belonged to the Ericales.

The complete chloroplast genome of *I*. *macrovexilla* (no. OK310515) and *I*. *macrovexilla* var. *yaoshanensis* (no. OK310516) had GC contents of 36.77 and 36.80%, which represented 152,437 and 152,286 bp, respectively. Both genomes contained a large single-copy region of 83,331 and 83,212 bp, a small single-copy region of 17,376 and 17,312 bp, and a pair of inverted repeats (IRA and IRB) of 25,865 and 25,881 bp, respectively. Both genome annotations revealed 88 protein-coding genes, 8 rRNA genes, and 37 tRNA genes. The phylogenetic analysis showed that *I*. *macrovexilla* and *I*. *macrovexilla* var. *yaoshanensis* were clustered closely together. These two specimens had close genetic relationships to *I*. *alpicola*, *I*. *fanjingshanica*, *and I*. *piufanensis*, but were distant from *I*. *guizhouensis* and *I*. *pritzelii* (Figure 1). These molecular results were consistent with the morphological classification described by Yu et al. (2007).

**Figure 1.**
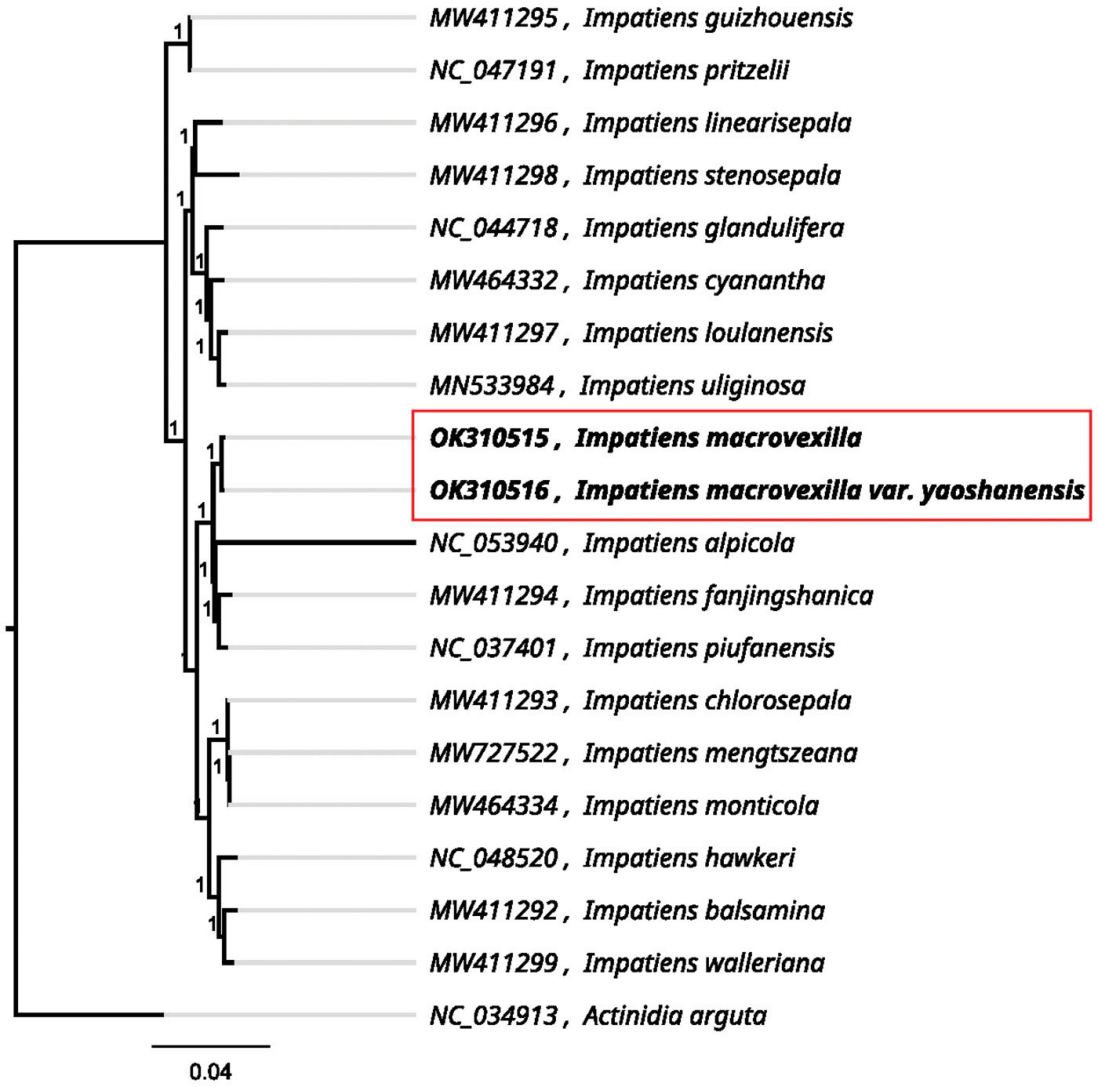
The maximum likelihood phylogenetic tree presenting the genetic relationships of *I*. *macrovexilla and I*. *macrovexilla* var. y*aoshanensis* among 17 other *Impatiens* species, using the generalized time-reversible model and the Shimodaira–Hasegawa test.

## Data Availability

The genome sequence data for *I*. *macrovexilla* and *I*. *macrovexilla* var. *yaoshanensis* that support the findings of this study are openly available in GenBank of NCBI [https://www.ncbi.nlm.nih.gov] under accession no. OK310515 and no. OK310516. The associated BioProject, SRA, and Bio-Sample numbers are PRJNA765922 and PRJNA765922, SRR16085899 and SRR16085898, and SAMN21592058 and SAMN21592059, respectively.
